# Why Self-Induced Pain Feels Less Painful than Externally Generated Pain: Distinct Brain Activation Patterns in Self- and Externally Generated Pain

**DOI:** 10.1371/journal.pone.0023536

**Published:** 2011-08-26

**Authors:** Yan Wang, Jin-Yan Wang, Fei Luo

**Affiliations:** 1 Key Laboratory of Mental Health, Institute of Psychology, Chinese Academy of Sciences, Beijing, China; 2 The Graduate University, Chinese Academy of Sciences, Beijing, China; University of Bologna, Italy

## Abstract

Voluntary movement generally inhibits sensory systems. However, it is not clear how such movement influences pain. In the present study, subjects actively or passively experienced mechanical pain or pressure during functional MRI scanning. Pain and pressure were induced using two modified grip strengthener rings, each twined with four crystal bead strings, with polyhedral beads to induce pain, or spherical beads to induce pressure. Subjects held one ring in the left hand and were either asked to squeeze their left hand with their right hand (i.e., active pain or pressure), or to have their left hand squeezed by the experimenter (i.e., passive pain or pressure). Subjects rated the intensity and unpleasantness of the pain sensation lower in the active procedure than in the passive one. Correspondingly, pain-related brain areas were inhibited in the case of self-generated pain, including the primary somatosensory cortex (SI), anterior cingulate cortex (ACC), and the thalamus. These results suggest that active movement behaviorally inhibits concomitant mechanical pain, accompanied by an inhibition of pain response in pain-related brain areas such as the SI cortex. This might be part of the mechanisms underlying the kinesitherapy for pain treatment.

## Introduction

Voluntary movement-generated, or “re-afferent”, sensory information is usually inhibited via an internal mechanism. Namely, when the brain initiates a movement, it also generates internal information, or corollary discharge, to cancel the influence of self-generated sensory information and prevent self-induced desensitization by inhibiting corresponding sensory systems. For example, neurons in the visual cortex have been shown to be inhibited by corollary discharges during saccades to suppress vision during eye movements (for reviews see ref [1], [2], [3]). Auditory researches in humans and animals revealed that phonation or sound-making actions inhibit the auditory system, thus protecting auditory sensitivity [4],[5],[6],[7],[8],[9]. Furthermore, active touch attenuates activity in the primary somatosensory cortex (SI) [10],[11], and active itch deactivates secondary somatosensory cortex [12],[13]. These studies support the view that active movement inhibits sensory systems.

As the inhibition in sensory systems during active movement seems to be an adaptive process, it is logical to assume that pain induced by self-generated movement should also be attenuated. Several studies have addressed this issue, but with inconsistent results. Some studies reported that active movement during pain application reduced laser-induced pain and attenuated the SI activity [14],[15], while other reports found no difference between the perceived intensity of self-induced (active) or externally induced (passive) thermal pain [16],[17],[18], though they did report interesting imaging results. For example, the SI and the posterior part of anterior cingulate cortex (pACC) were inhibited under self-generated pain but activated under externally generated pain, whereas perigenual ACC were mobilized in exactly the opposite way [17],[19]. The vermis of cerebellum were found less activated in active than in passive pain, under either normal condition [16] or thermal hyperalgesia [18]; while the activation of the anterior and the posterior insula, the secondary somatosensory cortex (SII) [19], and the mid cingulate cortex (MCC) [17] were independent of the pain application mode. Thus, behavioral findings regarding pain perception are controversial. In addition, very few studies investigated the behavioral effect of active movement upon the affective dimension of pain [15].

The current study was designed to test whether mechanical pain generated by active movement will be diminished compared with pain brought about by passive movement. Compared to thermal pain, mechanical pain is more often encountered in daily life, since most adults and children have experienced mechanical pain in stumbling, falling, knocking against chair or table, or being stabbed with a sharp object. With this study we would like (i) to prove that the effect is produced by self-related modulation rather than movement *per se*; (ii) to confirm that the effect can be detected also in mechanical pain condition. We would also like to examine whether this inhibition exists in both the sensory and the affective dimensions of pain [20]. Functional magnetic resonance imaging (fMRI) was employed to explore the possible brain mechanisms under this design. We hypothesize that the mechanical pain generated by active movement can also be diminished in both the sensory and the emotional dimensions. We also hypothesize that this effect is mediated by a neural matrix containing both the sensory and the affective pain pathways.

## Results

### Active movement inhibited pain

**Figure 1:**
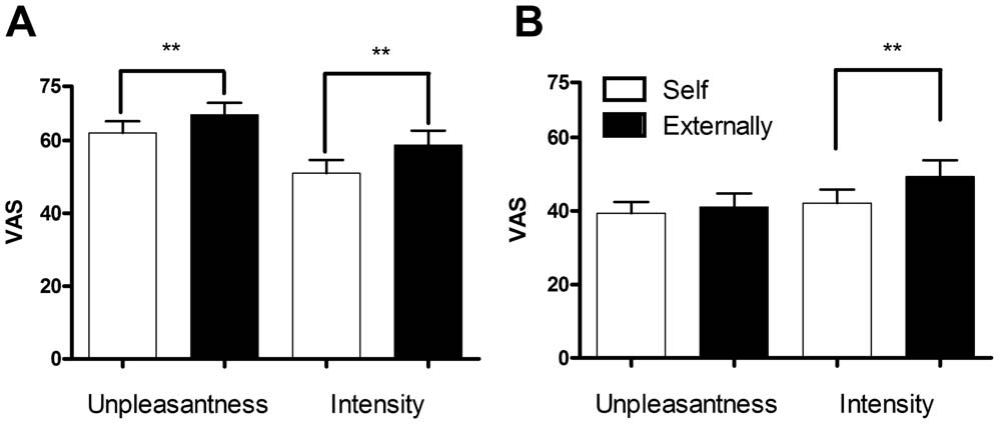
Behavioral ratings of pain stimuli. Ratings for unpleasantness and intensity of pain and pressure were compared in self-induced and externally induced conditions. (A) Both unpleasantness and intensity of self-induced pain were rated significantly lower than externally induced pain. (B) While unpleasantness ratings of pressure induced either way were not significantly different, the self-induced ones were rated significantly lower on intensity. VAS, visual analog scale. ***p<0.001, n = 25.

Self-induced pain was rated significantly lower than externally applied pain on both intensity (51.06±17.84 and 58.75±20.04, respectively, *t*-test, *p*<0.0001) and unpleasantness (62.12±16.21 and 67.15±16.21, respectively, *t*-test, *p*<0.0001), as shown in Figure 1A. Self-induced pressure was also rated significantly lower than externally applied pressure on intensity (42.08±18.97 and 49.37±21.97, respectively, *t*-test, *p*<0.001). However, the unpleasantness ratings of self- and externally induced pressure were not significantly different (39.32±15.53 and 41.09±18.08, respectively, *t*-test, *p*>0.05), as shown in Figure 1B.

### The brain recruited a large network to inhibit self-generated pain

**Figure 2:**
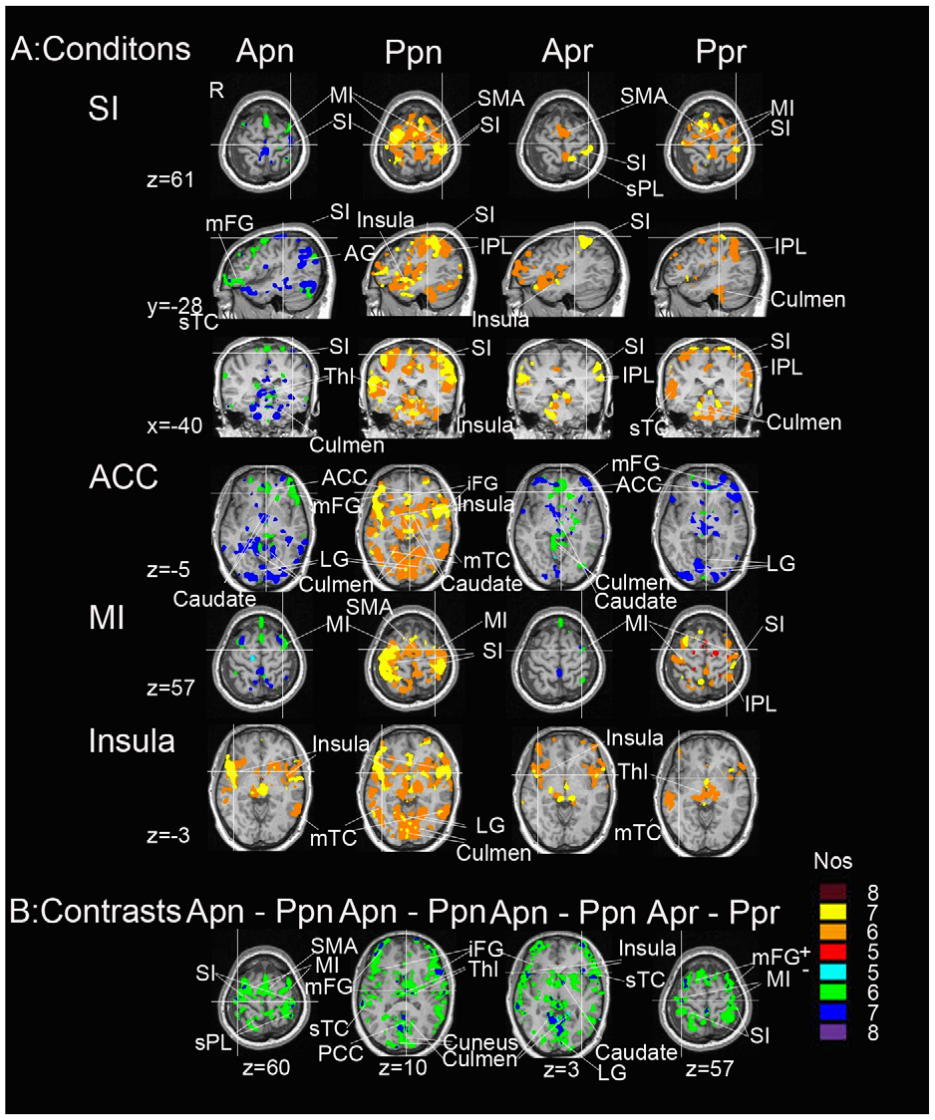
Regional brain activation in the conjunction analysis. A) Activation maps in the four conditions. Panels shown from left to right: active pain (apn); passive pain (ppn); active pressure (apr); passive pressure (ppr). The first three rows display the activation of SI, and the fourth to sixth rows show the activation of ACC, MI, insula in sequential order. B) Differences between pairs of conditions. The left three panels: active minus passive pain. The most right panel: active minus passive pressure. Nos, number of subjects. +: activation; −: deactivation. All data were corrected, *p*<0.05. R stands for right. SI, primary somatosensory cortex; MI, primary motor cortex; SMA: supplementary motor area; mFG, middle frontal gyrus; sTC: superior temporal cortex; Thl: thalamus; ACC, anterior cingulate cortex; AG: angular gyrus; LG, lingual gyrus; mTC, middle temporal cortex; iFG: inferior frontal gyrus; IPL: inferior parietal lobule; sPL: superior parietal lobule; PCC, posterior cingulate cortex.

Distinct activation patterns were found during the four conditions, as shown in Figure 2 and summarized in Table 1. Specifically, in reference to the squeezing movement of the right hand of the subjects, self-induced pain deactivated contralateral (i.e., left) SI, MI, superior temporal cortex (sTC) and middle frontal gyrus (mFG.), bilateral thalamus, ACC, caudate, angular gyrus (AG), lingual gyrus (LG) and culmen of vermis, and activated bilateral insula and middle temporal cortex (mTC). On the other hand, externally induced pain activated bilateral SI, MI, caudate, supplementary motor area (SMA), inferior frontal gyrus (iFG), insula, inferior parietal lobule (IPL), iFG, mTC, LG and culmen of vermis. Self-applied pressure produced activation in contralateral SI and superior parietal lobule (sPL), bilateral SMA, insula, IPL, LG and culmen of vermis, deactivated bilateral ACC and culmen of vermis, ipsilateral caudate and contralateral MI and mFG. Externally applied pressure also activated contralateral SI, but additionally activated ipsilateral mTC and thalamus, bilateral MI, IPL and culmen of vermis, while deactivated bilateral LG and ACC, and contralateral mFG.

**Table 1:** Brain areas affected by each condition.

Condition	Area	Side	x	y	z	Nos
*Self-induced pain*	SI (−)	L	−40	−28	61	7
	MI (−)	L	−36	−8	57	6
	AG (−)	B	−42/42	−61/−61	29/29	7/7
	Thalamus (−)	B	−10/10	−29/−29	3/3	7/7
	Culmen (−)	B	−14/13	−30/−30	−18/−18	8/7
	rACC (−)	B	7/−7	44/44	−5/−5	6/6
	Caudate (−)	B	−6/7	4/6	−3/−3	7/7
	LG (−)	B	−20/20	−87/−87	−5/−5	7/7
	Insula (+)	B	−39/42	13/9	−4/−4	6/7
*Externally induced pain*	SI (+)	B	34/−34	−36/−36	61/61	7/7
	MI (+)	B	−39/31	−28/−23	61/61	6/7
	SMA (+)	B	−6/6	−17/−17	61/61	7/6
	rACC (+)	B	−5/5	45/45	4/4	7/7
	IPL (+)	B	−40/36	−52/−48	52/52	6/7
	Insula (+)	B	−34/42	15/10	4/4	7/7
	Caudate (+)	B	−8/7	1/2	0/0	7/7
	Culmen (+)	B	−10/10	−44/−50	0/0	6/6
	LG (+)	B	−3/8	−69/−64	0/0	7/7
*Self-applied pressure*	SMA (+)	B	−8/6	−12/−6	61/61	6/6
	SI (+)	L	−37	−41	60	7
	Insula (+)	B	−39/41	14/13	−5/−5	6/6
	IPL (+)	B	−39/44	−45/−33	47/47	7
	Culmen (−)	B	−11/9	−41/−37	−6/−6	6/6
	rACC (−)	B	−4/6	46/43	−5/−5	6/6
	Caudate (−)	R	9	11	−6	7
*Externally applied pressure*	SI (+)	L	−35	−45	61	6
	MI (+)	B	−30/40	−25/−25	61/61	6/7
	IPL (+)	B	−56/56	−34/−34	35/35	6/6
	Culmen (+)	B	−7/6	−56/−56	−1/−1	7/7
	SMA (+)	B	−10/9	0/1	61/61	7/7
	Thalamus (+)	B	−15/15	20/20	−1/−1	6/6
	LG (−)	B	−11/27	−86/−86	−8/−8	7/7
	rACC (−)	B	−5/6	54/53	−5/−5	6/6
*Self-induced pain* – *externally induced pain*	SI (−)	B	−40/44	−33/−27	60/60	7/7
	MI (−)	B	−33/34	−11/−11	60/60	6/7
	SMA (−)	B	−5/5	−15/−15	60/60	6/6
	Culmen (−)	B	−8/8	−53/−56	3/3	7/7
	Thalamus (−)	B	−12/12	−16/16	10/10	7/7
	Caudate (−)	B	−6/9	1/16	3/3	7/7
	Insula (−)	B	−35/44	20/16	3/3	7/7
	LG (−)	B	−11/11	−71/−71	3/3	7/7
*Self-applied pressue* – *externally applied pressure*	SI (−)	B	−40/31	−33/−33	57/57	6/6
	MI (−)	B	−39/33	−25/−24	57/57	7/7

Nos: number of subjects in the conjunction analysis. Activation and deactivation are indicated by the “+” and “−”, respectively. All results are corrected for multiple comparisons, p<0.05. L, left; R, right; B, bilateral; SI, primary somatosensory cortex; MI, primary motor cortex; AG, angular gyrus; rACC, rostral anterior cingulate cortex; IPL, inferior parietal lobule; LG, lingual gyrus; SMA, supplementary motor area.

In comparison with passive pain, active pain deactivated bilateral SI, MI, SMA, culmen of vermis, thalamus, caudate, insula, sPL, iFG, mFG, sTC, posterior cingulate cortex (PCC), LG and cuneus. A contrast with passive press revealed less activation in the bilateral SI, MI and mFG in active press.

### Correlation between VAS and BOLD response

**Figure 3:**
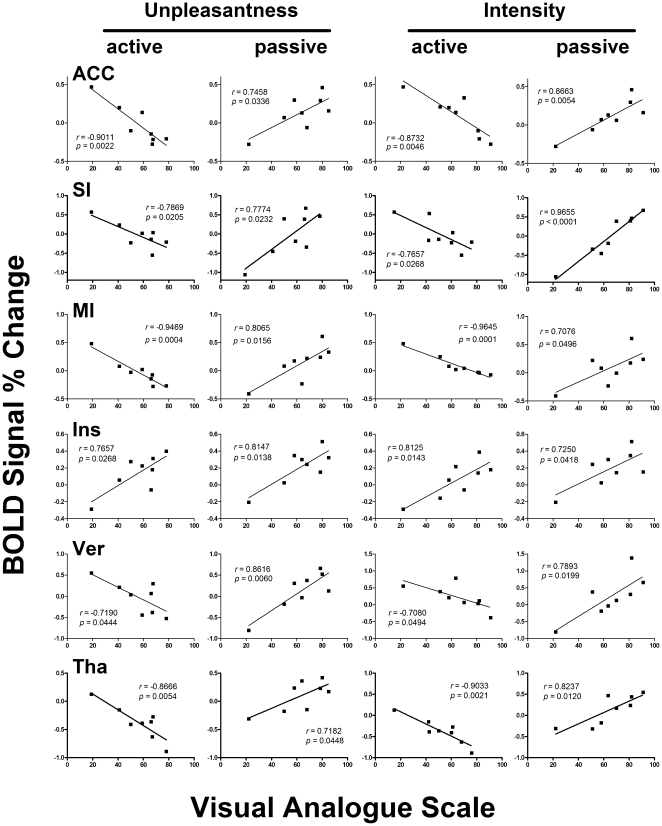
Correlation analysis between BOLD signal changes and pain ratings. Linear correlation analyses were performed for the BOLD signal changes in the ROIs with the unpleasantness ratings (left two columns) or pain intensity ratings (right two columns). Panels in the six rows (top to bottom) are scatter plots for the anterior cingulate cortex (ACC), the primary somatosensory cortex (SI), the primary motor cortex (MI), the insula (Ins), the vermis of cerebellum (Ver), and the thalamus (Tha). All ROIs except for the insula were negatively correlated with the ratings in active pain and positively correlated with ratings in passive pain. The Insula, however, were consistently correlated with all ratings in both conditions. All of the ROIs are based on functional and structural masks.

**Figure 4:**
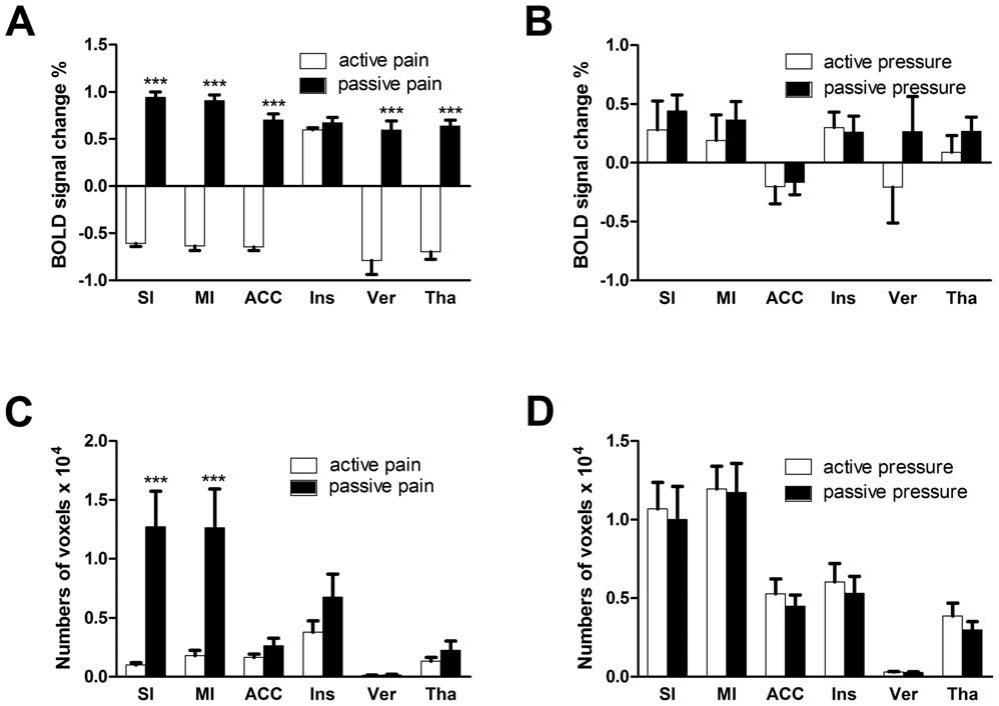
Statistics on functional brain activation. Mean intensities of BOLD signals and activated voxel numbers in the ROIs in active and passive pain conditions were compared. (A) In all ROIs except the insula, BOLD signal intensities were higher in passive pain than that in active pain condition. (B) No such difference could be found between active and passive pressure. (C) Passive pain activated significantly more voxels in the primary somatosensory and motor cortices. (D) No significant difference was observed between the two types of pressure.

To further clarify the relationship between BOLD signal intensity and pain ratings of intensity and unpleasantness, we performed correlation analysis between the ratings and BOLD activities in the whole brain, and found that the degree of activities in bilateral SI, MI, thalamus, ACC, culmen of vermis, and insula were significantly correlated with the pain intensity and unpleasantness ratings (data not shown). Then we selected these six regions as the regions of interest (ROIs) and compare the intensities and number of voxels in active and passive conditions. The results revealed that all ROIs except insula were negatively correlated with the intensity and unpleasantness ratings in active pain (see Figure 3, first and third column) while positively correlated with the ratings in passive pain (Figure 3, second and fourth column). For insula, BOLD signals were positively correlated with both ratings and in both active and passive mode (Figure 3, fourth row). The mean intensity of all voxels in all ROIs except insula in active pain were significantly less than that in passive pain (see Figure 4A), while the mean intensity of all voxels in active pressure were not significantly different from that in passive pressure (see Figure 4B). As regards to the number of voxels, only MI and SI were significantly less in active pain than that in passive pain (see Figure 4C), while no apparent difference of numbers was observed between the active pressure and passive pressure (see Figure 4D).

## Discussion

### Voluntary movement inhibits the pain it induces

The current study examined how pain perception is inhibited by voluntary movement. The results confirmed previous findings that self-induced pain is perceived as less intense and unpleasant than externally induced pain. Furthermore, we demonstrated that a large brain network was activated in the externally induced pain state, including the SI and caudate nucleus, which was consistent with previous studies [14],[21]. In contrast, during self-induced pain, pain-related brain areas including SI, thalamus, ACC and caudate nucleus were deactivated. These results suggest that suppression of the pain-related brain areas may underlie inhibition of pain by self-generated movement.

Our data support past studies that demonstrated movement-induced suppression of various sensory input, including visual, auditory and somatosensory. For example, saccades reduced the brightness of sight [22] and impaired detection of moving objects [23],[24]. Our results demonstrated analgesic effects of self-generated movement on both sensory and affective dimensions of pain, consistent with a previous study showing that the self-controlled pain was perceived as less intense and anxious than externally controlled pain [25]. Together with the literature, our results suggest that self-generated movement may suppress sensory systems in general and influence the affective dimension along with the sensory dimension of pain.

The influence of movement upon sensory systems is typically adaptive. Wurtz [3] proposed that saccades inhibit the visual system to avoid the perception of blur during movement. Others have suggested that sound-making movements may suppress the auditory system to prevent damage and maintain auditory sensitivity [6]. Both touch and itch inhibit the somatosensory cortex, preventing redundant information from ascending to the brain [12]. Similarly, self-generated movement may depress pain perception by blocking ascending nociceptive signals, which could suppress pain in situations where the organism needs to engage in fight-or-flight responses to survive.

### How does self-generated movement suppress pain perception?

The analgesic effect of voluntary movement may be due to inhibition of the pain matrix in the brain. One possible explanation for this effect is corollary discharge. Studies in the fields of vision, audition and somatosensation have demonstrated that when the central nervous system elicits a movement command, it also sends a copy, or corollary discharge, to inhibit the sensory systems. Similarly, electroencephalographic studies have found that self-generated movement suppressed the pain it induced and inhibited SI activity [15]. Although there is no direct evidence supporting corollary discharge for such inhibition, it is a commonly-accepted theory in the literature, and some researchers theorize that a source of corollary discharges may be cerebellum, from which the corollary discharges are sent to the parietal cortex [16]. Studies using fMRI support this theory by showing that self-generated movement deactivates sensory cortex [19]. Some researchers even speculate that corollary discharge contributes to the formation of the senses [26], and this view gains support from studies showing that self-generated touches are more likely to lead to object recognition [27].

However, the reason for the laterality of such inhibition was still unclear. It was reported that corollary discharges mainly inhibit sensory pathways contralateral to the voluntary movement in higher species [2], yet an imaging study found that voluntary movement deactivated the ipsilateral SI [19]. In fact, along with the significant deactivation found in the side contralateral to the squeezing right hand, there were scattered and borderline significant deactivation in the ipsilateral side (not reported in the table 1) in the present study. Thus it is possible that the active movement inhibited SI in the both hemispheres, yet imposed more sever effects on the contralateral side.

In addition to corollary discharge, another possible explanation for the suppression of pain by self-generated movement is the expectation of pain. Subjects in the current experiment engaged in the same movement under self- or externally generated pain conditions, but the expectation of pain was different. Specifically, for self-generated pain, subjects could anticipate quite accurately the time and intensity of the painful stimulation, unlike when pain was externally generated. It is well-accepted that certain expectations result in analgesia [28] while uncertainty can lead to hyperalgesia [29]. Thus, the analgesic effects of self-generated movement in this experiment might result from certain expectation in the self-generated pain condition.

In fact from a psychological perspective, the impact of self control on pain perception generally differs from that of being controlled. Previous studies have found that the analgesic effects appeared when subjects controlled noxious stimuli themselves [25], or viewed their pain-receiving hands directly or with a mirror [30]. These effects may be attributed to the emotional reappraisal of pain in such self-related conditions [25]. People with stronger self control or internal locus of control tend to have better performance in various tasks [31], [32] and be more tolerable to pain [33]. In clinical settings, active exercises and movements have been recommended as part of a comprehensive rehabilitation in patients with chronic pain. Our current results provide evidence that active movements produce less pain and help enhance pain tolerance, supporting the ‘paradoxical pain therapy’ in which patients are encouraged to use their injured body in spite of pain [34].

### Brain networks involved in analgesia during self-generated movement

The fMRI scans revealed two important results. First, many brain areas such as MI and LG that were not pain related showed distinct activation patterns in active and passive pain. This large-scale recruitment of brain regions (e.g., MI, iFG, mFG, AG, LG, caudate) may support the complex processing required by self-generated movement, including cognition, attention, motor planning, and execution. Second, bilateral insula were significantly activated in both the active and passive pain, while only slightly activated in active and passive pressure. This indicates that insula may be specific to stimuli properties and independent of ways of application.

Our results suggest that changes of the SI activity may be responsible for the analgesia induced by self-generated movement. Pharmacological manipulation of corticofugal modulation found that activation of cortical output in the SI enhanced response of the ventral posterior lateral nucleus of thalamus (VPL) to noxious stimulus, while inhibition of this area decreased the VPL activation [35]. Our behavioral study also confirmed that SI can modulate pain, with facilitation of acute pain while inhibition of chronic pain [36]. An electrophysiological study found that electrical stimulation of SI in rats attenuated dorsal horn neuronal response to noxious pinch [37]. Imaging studies discovered that self-generated movement could alleviate pain, possibly through the deactivation in SI, either contralateral [15] or ipsilateral [19] to painful stimulation. Although SI has not been consistently demonstrated to be associated with pain in earlier reports [38], recent imaging studies confirm the role of SI in coding pain intensity. A recent fMRI study found that activation of SI in human subjects was positively correlated with pain intensity [19]. Optical imaging studies also demonstrated that the intensity of optical intrinsic signals which indirectly reflects excitability of neurons in SI was correlated with the intensity of noxious stimulation applied to rats [39],[40]. Our current results showed that, under self-generated pain condition, the intensity of pain was negatively correlated with the BOLD signals of SI. Considering that SI neurons were largely activated by acute painful stimulation [41], and descending information flow greatly increased at this time [42], the SI activation may be the key factor for the perception of acute pain. Thus, the decrease of SI activity observed in the current report may reflect an involvement of this area in the inhibition of active movement-induced pain.

The role of ACC under such active and passive painful conditions is still not clear as apparent inconsistence was found between our results and previous findings. ACC generally contains three parts: perigenual or rostral ACC (rACC), mid ACC (mACC), pACC. For a long time, rACC has been considered to be a classical region in pain processing because it was persistently activated under externally applied painful stimulation. A previous imaging study showed that rACC was activated during pain caused by self-generated movement and deactivated by pain caused by externally generated movement, while pACC showed a reversed pattern, and mACC showed no difference [17]. Wiech et al. also found activation of ACC in the self-generated condition in an fMRI study investigating the neural correlates of analgesia associated with stimulus control [25]. In contrast, we found that rACC was deactivated in active pain and activated in passive pain, and no significant difference was observed in mACC and pACC. The reason for such inconsistency is not clear and may be caused by different ways of stimulus application or different time duration of movement. In the study of Mohr et al, the active movement was used to trigger the onset of the thermal pain stimulation, but with no control on the intensity (i.e., fixed) and duration (terminated by the investigator) of the stimuli. However, in our study, the active movement not only controlled the time course of the stimulation, but also the intensity of it. This makes the pain stimuli more controllable and hence more thoroughly ‘active’ for the subjects. That is probably the reason why our active pain activated less ACC area (no mACC or pACC mobilized) and the rACC were even deactivated. The negative correlation between ACC activation and pain rating further confirmed this finding.

In addition, rACC is part of ventral medial prefrontal cortex which process “self-related” information [43],[44]. Recent studies further support a pivotal role of ACC and PCC in the default mode network, showing activation at rest and deactivation under passive cognitive tasks [45],[46]. Thus, the role of rACC may be quite complicated. We consider that rACC is able to discriminate self and other agents, although the specific activation pattern may not be the same.

Recent studies have suggested that the basal ganglia nuclei, especially the caudate nucleus, might contribute to analgesia in addition to its well-known role in movement, although the pattern of activity is not consistent across studies. The caudate nucleus showed activation when subjects anticipated imminent painful stimuli [47] or tried to suppress pain sensations after pain onset [48],[49]. Conversely, it showed deactivation when the subjects did not try to suppress pain sensations [50]. Studies using acupuncture found that shallow needle punctures deactivated the caudate nucleus while deep punctures activated it [51]. Our results demonstrated that caudate nucleus was deactivated in the self-induced pain condition and activated in the externally induced pain condition. Thus, the caudate nucleus may also be involved in the analgesic effect of self-induced pain.

Previous studies found that vermis were activated in active pain yet less excited than passive pain under normal condition [16], or under thermal hyperalgesia [18]. In our study, correlation analysis of the brain activation with the ratings discovered that vermis was significantly and negatively correlated with the pain or unpleasantness ratings in active pain, but positively correlated with that in passive pain (see Figure 3B). Although cerebellum is mainly in charge of movement execution, subjects made the same movement under active and passive pain conditions in the previous studies [16],[18] and our in study. So the contrast of activities in vermis under active and passive pain unlikely came from movement execution. In our opinion, the less activation in previous studies and the deactivation of the culmen of vermis in our study are probably because that the vermis receives some top-down intervention or cognitive control. Combined with past researches, we consider that culmen of vermis may play a role in analgesia during pain-inducing voluntary movement. The reason that MI was deactivated in active pain is not so clear. Possible explanations may be that MI received inhibition from higher processing areas via motor planning areas [8], or from cerebellum via parietal areas [16],[52].

It was reported that anterior insula showed almost the same activation pattern in both application (active and passive) modes in the previous studies [18],[19]. The present study confirmed this (see Figure 3, fourth row, and Figure 4C), so it is unlikely to be involved in the inhibition of self-generated pain.

### Conclusion

When self-generated movement induces pain, the brain compensates to induce analgesia by inhibiting the SI, thalamus and pain-related areas, such as the caudate nucleus and limbic system. We assume that this inhibition may be due to corollary discharge or expectation of pain. This phenomenon could be considered adaptive for survival because it allows engagement in fight-or-flight response, or other necessary activities even at the threat of hurting ourselves.

## Materials and Methods

### Ethics statement

Written informed consents were obtained from all subjects prior to the experiment. The study was conducted in accordance with the Declaration of Helsinki, and it was approved by the Institutional Review Board of the Institute of Psychology, Chinese Academy of Sciences.

### Subjects

Twenty-five healthy right-handed college students (14 male and 11 female, aged 24.3±0.3 years) volunteered to participate in the behavioral tests. Eight of these subjects (5 men and 3 women, aged 22.9±0.8 years) also participated in fMRI scanning.

### Mechanical stimulation apparatus and procedure

**Figure 5:**
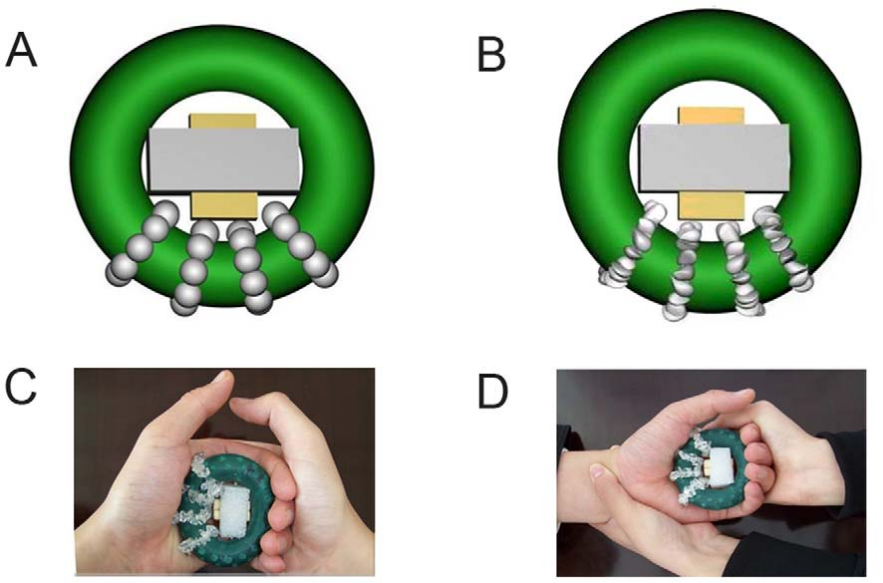
Schematic diagram of the pressure and pain stimuli and experimental conditions. (A) Pressure ring with spherical beads. (B) Pain ring with polyhedral beads. (C) Self-induced movement, in which the subject squeezes his own left hand with his right hand; the pain ring is pictured. (D) Externally induced movement, in which the experimenter squeezes the subject's left hand; the pain ring is pictured. Within the rubber ring was the cross holder, consisted of the yellow short arms and the grey long arms.

Two rubber grip strengthener rings (HJ-WL03-RG, Xinjia Plastics, Guangdong, maximum force 10 kg), were modified to induce pressure or pain sensations. The inner and outer diameters of the rings were 34.5 mm and 67.0 mm, respectively. Four crystal bead strings were wound around each ring and fixed by cyanoacrylate adhesive so that the beads were evenly spaced over a quarter of the ring. The crystal beads were 8 mm in diameter, and were either spherical to induce a mechanical pressure sensation (the pressure ring, see Figure 5A), or polyhedral to induce a mechanical pain sensation (the pain ring, see Figure 5B). A cross holder was placed in the middle of each ring with the long arms braced firmly inside while the short arms served as a restriction for maximal grip force. Squeezing action was trained to the extent that the inner side of the ring just touched the short arms to maintain consistent squeezing force. The pain threshold was accessed by squeezing slowly of the pain ring until pain occurred, and the inner short diameters were recorded and averaged among three trials for each subject. The short arms of the cross were adjusted so that it was slightly shorter than the pain threshold, so that pain can be induced consistently in each squeezing. We confirmed in a pretest that the pressure ring with the same force could only induce pressure. The moderate nature of the strengthener made sure that apparent fatigue be avoided during the experiment.

**Figure 6:**
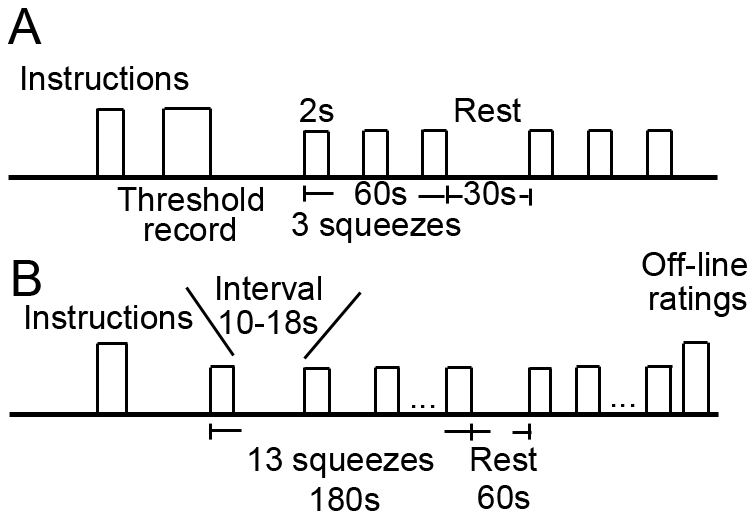
Procedural timeline for behavioral tests and fMRI scanning. (A) Subjects underwent self-induced and externally induced squeezes in the pressure and pain conditions in a within-subjects design. Squeeze for 2 seconds/trial was repeated three times in 60 seconds. The interval between conditions was 30 seconds. All conditions were repeated three times, and condition order was randomly determined and counterbalanced. (B) During fMRI scanning, squeeze was repeated thirteen times in each condition with 10–18 seconds between trials. Each condition lasted for 180 seconds, and the interval between conditions was 60 seconds. Subjects gave off-line VAS ratings after scanning was completed.

Subjects were instructed to sit in an armchair, rest their elbows on the arms of the chair and relax their left hand with the palm facing upward. They were asked to avoid making any movements unless instructed to do so. One of the two test rings (pain or pressure) was then placed on their left palms with the beads contacting the palm. The subjects were asked to squeeze their left hands with their right hands (i.e., active pain or pressure, see Figure 5C), or the observer squeezed the left hand of the subject (i.e., passive pain or pressure see Figure 5D). This resulted in four conditions which were tested in a within-subjects design and in a randomized order. In the behavioral test, each condition consisted of three trials of 2-second squeeze in 60 seconds with a 30-second interval between two conditions (Figure 6A). In fMRI scanning, there were thirteen trials of 2-second squeeze in each condition that lasted for 180 seconds with a random inter-trial interval of 10–18 seconds to reduce expectation and a 60-second resting time between two conditions in fMRI scanning (Figure 6B). Considering that the movements involved both hands (subject squeezed the left hand with his right hand) in the active condition and only one hand (the experimenter squeezed the left hand of the subject) in the passive condition, the subject was required to squeeze another ring (without crystals) in his right hand in the passive condition to counterbalance the bias.

### Experimental protocol

Pain thresholds were measured for the 25 subjects prior to starting the experiment. Subjects then underwent the mechanical stimulation procedure (described above), rating their perception of the intensity and unpleasantness of pain or pressure using the visual analogue scale (VAS) after each trial. The VAS consisted of a series of faces slowly changing from smile to cry on the front side and a 100-mm horizontal line on the back side. Subjects were told that the smiling face indicated “no pain” or “no unpleasantness” and the crying face meant “most pain imagined” or “most unpleasant” in the rating of intensity or unpleasantness of pain, respectively. In the rating of pressure, however, the smiling face indicated “least pressure” and “most pleasant”, while the crying face meant “most pressure” and “most unpleasantness”. The subjects indicated the location which best fit their perception by moving a cursor on the scale, and the observer recorded the corresponding numbers from the back for statistical analysis.

Eight of the 25 subjects underwent fMRI scanning and participated in the squeeze tests. Voices of two verbs (‘go’ and ‘stop’) cued the beginning and end of each squeeze. Subjects were instructed to keep their eyes open, relax their mind, pay attention to the rings and remain as motionless as possible throughout the experiment. Immediately after MRI scanning, subjects were asked to give off-line VAS ratings of the average pain intensity and unpleasantness. We did not ask the subjects to give online ratings because this might distract subjects' attention and induce possible disturbance from movement either of speaking or counting fingers for intensity or unpleasantness of pain. We also verbally ascertained whether the intensity or unpleasantness of the stimuli changed during the experiment, and no subjects reported any obvious change in either measure. Since all subjects were well trained for the squeeze test before the beginning of the behavioral test and the fMRI scanning, they are pretty steady-handed in the operation and thus equivalent results can be expected between these two tests.

### Image acquisition

The experiment was performed using a 3.0 T Trio MRI scanner with a standard head coil. Functional T2*-weighted blood oxygen level-dependent (BOLD) images were acquired using a gradient echo planner imaging sequence (TR/TE/FA: 2000 ms/40 ms/90°; FOV: 220×220 mm2; Matrix: 64×64 pixels). Twenty consecutive axial slices (thickness 5 mm, gap 0.5 mm, voxel size = 3.438×3.438×5.5 mm3) were acquired for each brain volume. For anatomical reference, a magnetization prepared rapid gradient echo imaging (MPRAGE) sequence was selected and the images (96 sagittal slices, voxel size = 1.7×0.86×0.86 mm3) were acquired. Another set of turbo spin-echo (TSE) T1-weighted images (20 axial slices, voxel size = 0.43×0.43×5.5 mm3) with identical position to the functional acquisition images was obtained for image registration.

### MRI processing and statistical analysis

MRI processing and analyses were performed using AFNI (Medical College of Wisconsin, Milwaukee; http://www.afni.nimh.nih.gov/afni/index.shtml) [53]. Slice time and motion were corrected, and reconstructed data were realigned, spatially normalized, and smoothed with a full width half maximum (FWHM) of 8 mm. Normalization was manually performed with verification from a neuroanatomist. For each condition, preprocessed MRI data were analyzed statistically using a general linear model (GLM). Multiple-correction was applied with AlphaSim. First the program 3dFWHM was used to get the least value for each condition, and this value was then used in the program AlphaSim with a cluster radius of 1.1 mm (voxel size changed to 1×1×1 after normalization) to generate the least size of clusters to make the significance of result below 0.05. After acquiring the least number of clusters, we used interactive 3Dcluster program on the panel of AFNI to get significant clusters after correction. For group analysis, a conjunction method was employed to find overlapping brain regions in statistical activation maps within individual subjects (for more details of the conjunction analysis see Heller et al [54]).

The fMRI-behavior relationship was assessed by Pearson correlation in each experimental condition (active vs. passive). Deconvolved impulse response function (IRF) from all subjects was concatenated into a single dataset. Then 3dfim was used to calculate the correlation between fMRI data and behavioral ratings. Regions of interest (ROIs) were defined by the *p* values of correlation analysis between whole-brain IRFs and pain ratings, as well as the relevance of these regions to pain processing. Within each ROI, a further correlation analysis was performed between the mean IRFs and behavioral measures. For more details please see ref [55], [56], and [57].

Behavioral data were summarized as means ± SEM. Paired *t*-test was calculated with *GraphPad Prism* 5 (GraphPad Software, Inc) to examine the difference between self and external conditions in the intensity ratings, unpleasantness ratings, as well as the BOLD signal changes.
